# Genomic Approaches to the Genetics of Alcoholism

**Published:** 2002

**Authors:** Marissa A. Ehringer, James M. Sikela

**Affiliations:** Marissa A. Ehringer, Ph.D. is a research associate, and James M. Sikela, Ph.D., is an associate professor, both in the Department of Pharmacology and Human Medical Genetics Program, University of Colorado Health Sciences Center, Denver, Colorado

**Keywords:** genetic theory of AODU (alcohol and other drug use), AOD dependence potential, alcoholic beverage, genome, gene expression, genetic mapping, DNA, mRNA, genetic correlation analysis

## Abstract

When studying complex diseases such as alcoholism that develop as a result of numerous genetic and environmental factors, researchers can use the sequence data that have become available both for the human and for animal genomes. For these analyses, investigators are being aided by efforts to identify and characterize functionally relevant DNA sequences in the entire genomic DNA sequence—a process called annotation. Various bioinformatics and annotation tools can help in this enterprise. These include four primary approaches: (1) precomputed, annotated public Web sites that provide a plethora of information; (2) in-house analyses from which users can choose the appropriate analyses for their purposes; (3) Web-based annotation systems that analyze a user’s DNA sequence; and (4) private resources that provide access to annotated genomic sequences at cost. In addition to careful study of the DNA sequence for clues about function, expression studies of mRNA levels using gene chips provide information about the activity levels of thousands of genes that may vary in different tissues, different animals and people, or under different environmental conditions.

Since the 1980s, researchers have attempted to identify genes that underlie various diseases. Initially, these efforts focused mainly on relatively rare genetic diseases—such as cystic fibrosis and Huntington’s disease—that because of their simple inheritance patterns were likely to be caused by only one gene. Using family studies to search for certain DNA segments (i.e., markers) that occurred only in people affected by these diseases, researchers successfully identified the underlying genes. More recently, the field of genetics has entered an era in which much of the focus has shifted to the study of more complex disorders—such as cancer, diabetes, hypertension, schizophrenia, and alcoholism—that are believed to develop as a result of a combination of numerous genetic and environmental factors.

The genetic study of complex diseases poses challenges that are not typically associated with single-gene disorders. For example, complex diseases are generally more common than single-gene disorders, tend to involve multiple genes, also include significant environmental factors, and are associated with a variety of characteristics and behaviors, or phenotypes, that are not simple to describe. To separate the multiple genetic and environmental components that underlie these diseases, researchers have developed more sophisticated methodological and statistical techniques. These techniques exploit knowledge about the inheritance of chromosomes, about the reorganization of genetic material that occurs during the generation of eggs and sperm (i.e., recombination), and about the analysis of quantitative traits—characteristics, such as height or intelligence, that vary along a continuum in the population. Based on this knowledge, investigators can identify specific DNA markers that are linked to complex diseases, thereby delineating regions within the genetic material of the cell that are likely to contain genes which contribute to complex phenotypes. These regions are called quantitative trait loci (QTLs). Many different alcohol-related QTLs have been identified in recent years, both through family studies of human populations and through research in model organisms, such as mice, rats, and flies.

Concurrent with the efforts to identify QTLs in alcohol studies, a vast array of bioinformatics resources have become available as the result of projects to decipher the entire genetic information (i.e., the genome) of various organisms. After the March 2000 publication of the genome sequence of the fruit fly *Drosophila melanogaster*, a draft of the entire DNA sequence of the human genome was completed, and in December 2002, public access to a draft sequence of the complete mouse genome became available (Waterston et al. 2002).

A major challenge facing genome researchers is to make sense of the vast amounts of “raw” genome sequence data that are being generated. For example, only about 1.5 to 2 percent of the genome is thought to represent gene-coding regions—that is, regions that specify what protein product will be produced. Most of the DNA in between the genes is noncoding and its functions are not fully understood. Some of those DNA sequences serve to regulate gene expression—where, when, and to what degree the gene is active. In addition, at least in higher organisms, genes typically consist of different pieces called exons, which contain the actual coding regions and which are interrupted by noncoding sequences called introns. When a gene is expressed, an RNA copy of the gene is produced that is then processed so that intron regions are removed and exon regions spliced together, forming a mature mRNA^1^ that carries the information to produce a specific protein product.

To identify genes associated with a disorder or characteristic, one must be able to distinguish genes from non-coding sequences, exons from introns, and regulatory signals from other DNA sequences. The process of identifying these various DNA sequences and signals within the vast amount of genomic DNA is called annotation. In recent years, a variety of bioinformatics tools have become available for annotating the increasing amounts of raw sequence data for various organisms. Likewise, additional projects are in progress to identify subtle genetic differences called single-nucleotide polymorphisms (SNPs) that exist among individuals in human populations and which can serve as additional DNA-based markers for analyses of the human genome.

Based on the information from the draft sequence of the human genome, researchers have estimated that the total number of human genes is approximately 30,000 to 40,000 (Claverie 2001). Alterations in the coding regions of genes themselves can account for some of the genetic differences among individuals and also can be responsible for the development of various disorders. However, individual differences as well as various genetic diseases can also result from other changes in the genome, including differences in the sequences that affect how a gene is regulated (e.g., how the information within a gene is spliced together or how the protein products of genes are assembled and modified). Annotating the genome sequence and investigating variability in the genomic sequence using the data coming out of the various genome-sequencing efforts require advanced bioinformatics tools. Many such programs and resources are now available and more are under development. Alcohol researchers can use these tools to identify potentially important genetic variations, such as gene-coding region changes or gene regulatory differences that may influence alcohol-related phenotypes.

This article provides an overview of the status of various genome projects, reviews several technologies used to determine differences in gene structure and expression, and summarizes some of the applications of these tools and technologies in the alcohol research field. Because full explanations of these highly technical analytical methods would go beyond the scope of an overview article, some of the information presented here will necessarily be abbreviated and simplified. The reader is referred to other sources, such as the numerous Web sites listed in tables 1 and 2, for more specific information on the tools discussed in this article.

## Genome Projects: Status of the Sequencing Efforts

In February 2001, researchers presented two draft versions of the human genome. One version was generated by researchers participating in the publicly funded Human Genome Project (HGP), which had been initiated in 1988. The HGP investigators first generated a rough map of the genome and then determined the exact DNA sequences of individual segments of that map. The second draft of the genome was generated by Celera, a genomics company established in 1998. Celera investigators employed a slightly different strategy in which they determined the DNA sequence of randomly selected DNA segments—a process called shotgun sequencing—and combined this information with data available from the HGP to obtain an assembled human genome sequence. Both groups published the results of their work the same week in the scientific journals *Nature* and *Science*, respectively (Lander et al. 2001; Venter et al. 2001). Interested scientists can access both data sets online, although access to the Celera database must be purchased. The availability and utility of these resources offer alcohol researchers unprecedented opportunities for identifying genes and molecular mechanisms that contribute to the development of alcoholism.

Numerous investigators have now mapped and sequenced the mouse genome (Waterston et al. 2002). Geneticists expect that the high degree of similarity between the human and mouse genomes will facilitate navigation back and forth between model organism and human, both at the DNA level and at the level of analyzing the functions of specific genes. Currently, nearly completed sequences for the mouse have been generated and assembled by both public and private (Celera) mouse genome-sequencing efforts. The data produced by the public sequencing effort are available online from the University of California at Santa Cruz, the National Center for Biotechnology Information, and Ensembl, which maintain public genome databases in the United States and United Kingdom. These resources for the mouse genome have been designed to mirror similar genomics resources for the human genome, located at the same Web sites, described below.

## Bioinformatics and Annotation Tools

Now that the DNA sequences covering the entire genomes of many different organisms have become available, the development of methods to decipher the roles of the genes residing within the DNA sequences has become a challenging but important task (Stein 2001). Even after the draft versions of the human genome have been completed, the functions of the great majority of the 3 billion nucleotides making up the genome remain unknown. To meet the challenges of identifying genes and other features in genomic sequences, a variety of bioinformatics tools have been developed.

As mentioned earlier, the part of a gene that actually encodes a gene product (typically a protein) is located in one or more exons. Because of their importance to genetic disease processes, it is crucial to identify exons within genomic DNA sequences (e.g., within QTLs that have been identified through other analyses). Several tools are available for this purpose, including various gene prediction programs, tools that search for similarities (i.e., homologies) between sequences, and other resources for detecting elements related to gene expression that are conserved across species.

Most of these tools are based on the hypothesis that because many important cellular functions and processes are highly conserved among diverse organisms, certain DNA sequences also have universal functions. For example, sequences signaling the beginning of a gene and splicing signals will be very similar for all genes in an organism and for different organisms. Accordingly, computer programs can search DNA sequences for the presence of potential start and splice signals and thus predict the location of exons. The various exon prediction programs available use three main algorithmic approaches that differ slightly among each other.^2^ The most effective means of identifying DNA sequences that are likely to represent true exons is to use multiple exon prediction programs to analyze a region of interest, because researchers can then focus on those regions that are predicted by more than one program (Fortna and Gardiner 2001). Identifying other DNA sequences with known functions that are highly conserved can provide additional evidence that sequences identified as potential exons are indeed parts of actual genes.

Alcohol researchers can use four primary approaches, discussed in the following sections, to identify genes that are present in alcohol-related QTLs:

- *Precomputed annotated public Web sites* can provide all of the necessary information for many applications and require minimal computer expertise.
- *In-house analyses* allow users to choose specific programs for identifying exons or other genomic sequence landmarks; these analyses can be useful for researchers with bioinformatics and computer expertise.
- *Web-based annotation systems* allow users to enter a genomic DNA sequence of interest and receive results via e-mail or a Web server.
- *Private resources* provide access to annotated genomic sequence at a cost.

### Public Precomputed Resources

Currently, three primary public pre-annotated databases exist: the University of California Santa Cruz (UCSC) browser Golden Path, the National Center for Biotechnology Information (NCBI) Map Viewer, and Ensembl. (Web sites for accessing these and other tools used in genomic analyses are listed in table 1.) Although they display data in slightly different ways, the three databases feature similar types of information. Known genes are illustrated on a map of the genome, along with such annotations as known mRNAs, gene predictions, and various conserved sequence elements. Numerous hypertext links allow easy access to additional information about the genes, including quick access to references of published papers.

#### Golden Path

**Figure 1:**
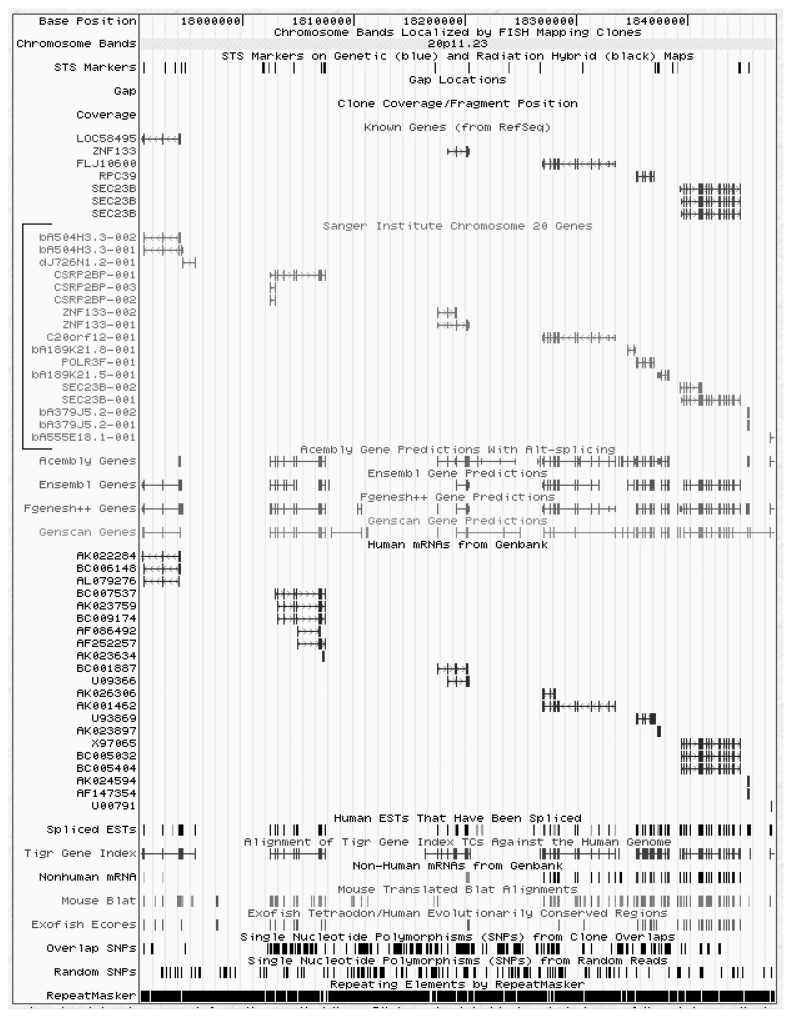
Example of a search result obtained with the University of California Santa Cruz (UCSC) human genome browser, Golden Path, showing known genes, predicted genes, and various other details about the genomic DNA region examined. For known genes (see bracket) the name of the gene serves as a hypertext link to additional information about the gene. From there, one can access other databases to learn about the function of the gene, mRNA expression data, homologous genes in other organisms, and other relevant information.

The UCSC genome browser has been developed emphasizing ease of use and simplicity. For researchers with limited experience in bioinformatics and genomics, this annotated version of the human genome may be the best place to start. It includes a user’s guide that describes the different types of information presented. The browser feature allows for a keyword search to find genes, specific chromosomal locations, markers, and other landmarks. The search results immediately point to a region of interest in the genome (see figure 1), provide information about numerous known features of that region, and offer access to additional information about known genes and other sequence elements.

#### Map Viewer

**Figure 2:**
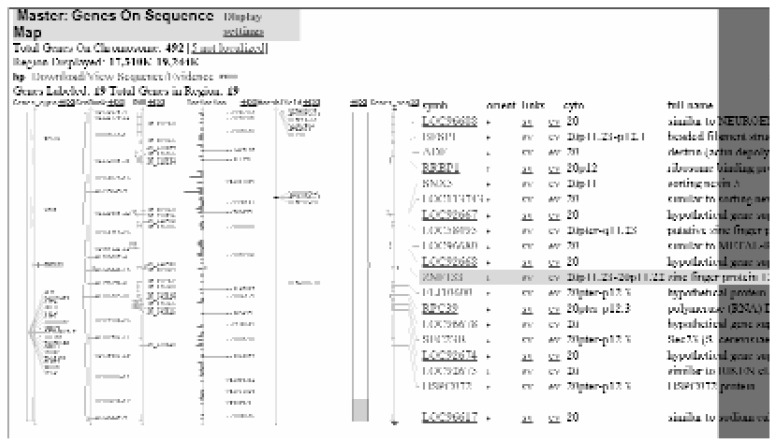
Example of a search result obtained with the National Center for Biotechnology Information (NCBI) Map Viewer. The map displays information about known genes, marker locations, and different types of maps of the human genome.

For investigators familiar with the Golden Path annotation and the types of data available, the NCBI Map Viewer also provides comprehensive annotation of the human genome (figure 2). Although somewhat more complicated to navigate, this browser provides additional resources not found on Golden Path. For example, it can simultaneously display up to seven different maps of the same DNA region, providing different types of information on each. A Help document on the home page gives details about all of these different types of maps as well as about how to best display the information. As with the Golden Path site, hypertext links are provided at nearly every step to connect the investigator to the latest information about genes, markers, and other sequence elements.

#### Ensembl

**Figure 3:**
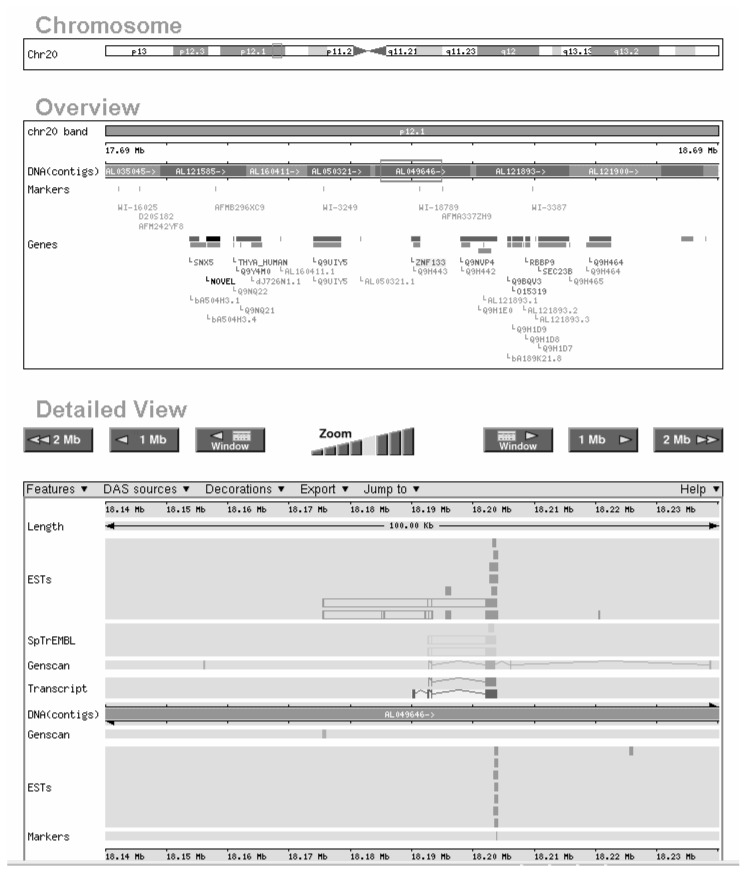
Example of a search result obtained with the Ensembl human genome browser. The map shows known genes, predicted genes, marker locations, and other genomic DNA details.

Like the other two sites, Ensembl provides map views of the genome (see figure 3). In addition, Ensembl generates specific reports for each search that is conducted, including such information as a list of the aliases under which a given marker might appear in other databases. A search for a specific gene generates an Ensembl Gene Report that lists information about the gene’s location and function, methods used to predict the gene, links to other databases, and other relevant information.

### In-House Methods

For researchers who want to perform their own analyses without relying on public databases, two primary programs, or workbenches, are available to facilitate annotation and analysis. These workbenches are called Genotator and Alfresco. Overall, these programs are very similar, although Alfresco can be downloaded onto a Macintosh, PC, or UNIX computer, and Genotator must be installed on a UNIX computer. The results of various types of sequence analyses, which are described in the following sections, can be plugged into these workbenches for combined analysis.

Conducting their own analyses of genomic sequences holds several advantages for researchers. First, the public annotated sites are only updated every few months, so the annotations may not include the most recent information. Other databases, however, are updated daily and can be accessed during an in-house analysis for up-to-the-minute examinations. Second, at present the annotations found on public Web sites are generated using only selected exon prediction programs, whereas specialized workbenches allow for incorporation of many other programs. Third, the flexibility of a workbench also allows inclusion of additional types of analyses, depending on the interest of the investigator. Finally, the browsers associated with these two popular workbenches have been designed to simplify analysis of the results.

**Figure 4:**
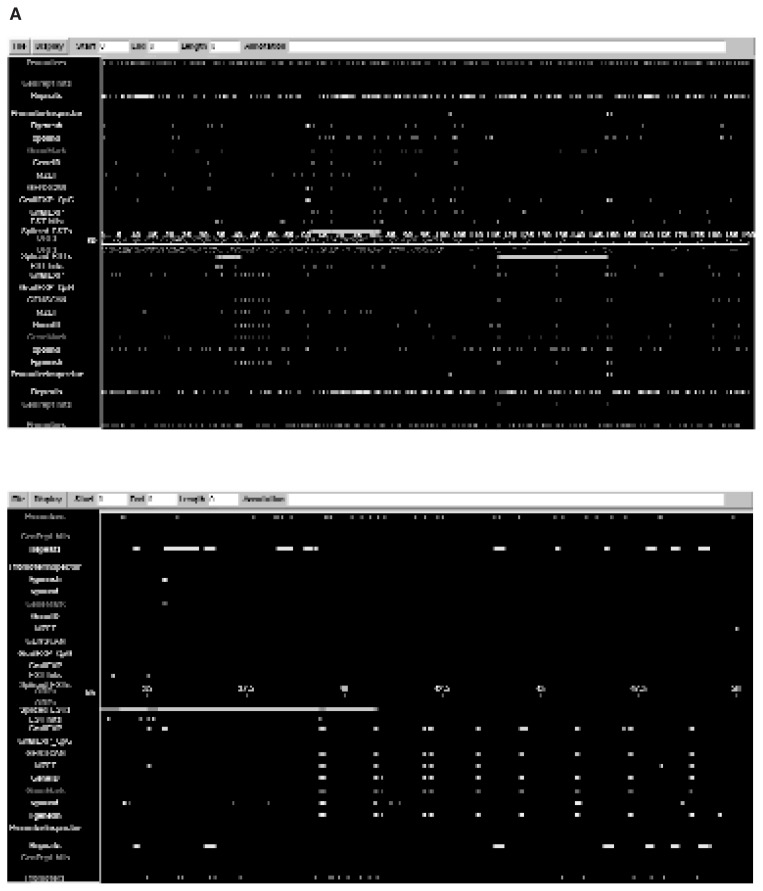
Example of search results obtained with a series of analyses that were entered into the Genotator browser. (A) A color-coded window depicts the results from the multiple software analyses completed. The display shows the locations of expressed sequence tags (ESTs), results from gene prediction programs, promoter predictions, and protein peptide similarities. (B) A closeup of an area of the display shown in Figure 4A, revealed by using the scroll bar in the left corner of the screen. By clicking on these regions with the mouse, one can view the DNA sequence and gain additional information.

The following sections describe three classes of analyses that can be performed on genomic DNA sequences—homology searches, exon prediction, and other analyses. (For a listing of these tools and information about them, see table 2.) Results from each of these analyses can be incorporated into the Genotator or Alfresco browser for simplified viewing of the combined results (see figures 4A and 4B) and for further information.

#### Homology Searches

An important step in analyzing unknown DNA sequences is to search for similarities, or homologies, with already known sequences because such homologies can give an indication of the function of the unknown sequence. For example, genes with similar functions (e.g., binding to DNA to regulate gene expression) often are characterized by specific DNA sequences. Similarly, related genes of humans and other organisms—and even noncoding DNA sequences—are often highly homologous, and identification of a known mouse DNA sequence with homologies to an unknown human sequence could suggest the function of the human sequence.

To identify homologies, one must first identify and block DNA regions in which short sequences are repeated several times. These repeats, which often are very similar among organisms but are so common that they provide no substantial information, can interfere with the analysis of other homologies. One program to identify and then “mask” these repeats is called Repeat Masker. The sequences that remain after the repetitive sequences have been masked are then compared with other databases, such as the NCBI EST database^3^ and protein databases. The output from these searches can be processed and then incorporated into the Genotator or Alfresco browser (Harris 1997, 2000).

#### Exon Prediction

Another approach for determining whether newly identified sequences contain a gene is to identify potential coding sequences, or exons. Several exon prediction programs (see table 2) can be used to analyze genomic sequences, including:

- Xpound, one of the earliest gene-finding programs (Kamb et al. 1995)
- MZEF (Zhang 1997, 1998)
- GrailEXP, which can model more complicated gene structures, such as one gene embedded within an intron of another, and can predict exon boundaries more accurately than other programs (Xu et al. 1997)
- GeneID, which has been designed with a hierarchical structure in which several steps are performed consecutively to help identify exons and genes (Parra et al. 2000)
- Eukaryotic GeneMark.hmm version 2.2a
- GENSCAN (Burge and Karlin 1997)
- Fgenesh (Solovyev and Salamov 1997).

#### Additional Analyses

Several other tools can be used to analyze unknown DNA sequences. For example, researchers can look for “open reading frames”—areas of DNA that could encode at least short proteins. One program that allows this type of analysis is called ORF Finder. To identify promoters—short stretches of DNA that regulate gene expression and are typically adjacent to the start site of a gene—researchers can use programs such as Neural Network Promoter Prediction (NNPP) (Reese and Eeckman 1995) or PromoterInspector (Scherf et al. 2000). Finally, investigators may wish to identify CpG islands, DNA regions with a characteristic composition of DNA building blocks, which are commonly located near the starting regions of genes. One program available for this purpose is the previously mentioned GrailEXP package (Xu et al. 1997).

### Web-Based Annotation Services

In addition to comparing genomic sequences with precomputed DNA analyses and annotations available through various Web browsers, researchers can submit their sequences via a Web site for custom analysis. This approach would be useful primarily to researchers who through in-house sequencing have obtained genomic DNA sequences from those regions of the genome that remain as gaps in the public database. Three major Web-based resources allow investigators to submit genomic sequences to a server and receive analysis results via e-mail, namely:

- *The NIX application*, which includes seven exon prediction programs as well as other applications and allows the user to incorporate additional annotations that could be useful for giving presentations
- *The RUMMAGE server*, which is more comprehensive than NIX but not as easy to use (Fortna and Gardiner 2001)
- *The Gestalt system*, which also allows for incorporation of additional annotations.

Some of these services use analysis programs that have not been incorporated into the pre-annotated genome browsers.

All of the human genome annotation options described so far in this article, including the pre-annotated, in-house, and Web-based systems, have been evaluated in a review by Fortna and Gardiner (2001).

### Private Annotation Services

**Figure 5:**
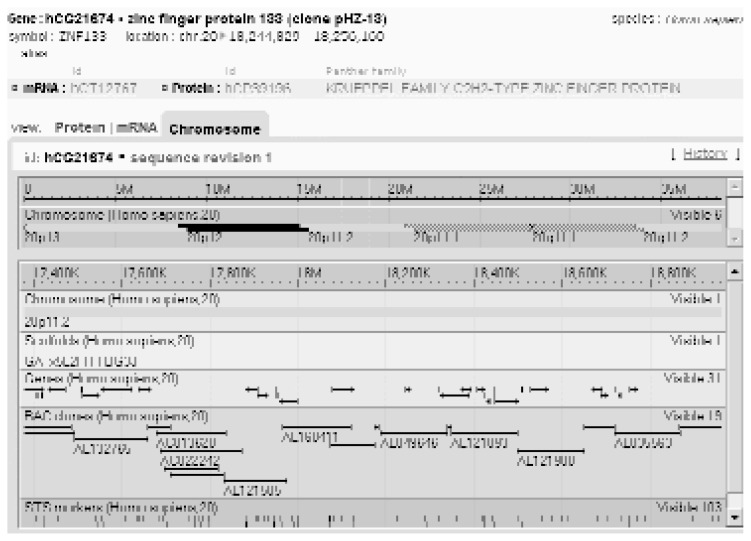
Example of a search result using the human genome browser developed by Celera Genomics. The map includes many annotations similar to those in the public domain, as well as additional results generated by Celera from analyses of gene predictions and information about novel genes that may be related to known genes.

In addition to the free resources described above, a private company—Celera—provides a version of the annotated human genome at a significant cost. Celera has developed a detailed, methodical approach for identifying genes that incorporates into its database various types of information about all genes identified by the company (figure 5). Celera also has analyzed the mouse genome and can provide the data as an assembled and annotated genome database. Additional information about Celera’s databases is available on the company’s Web site.

## Expression Studies

As mentioned earlier, alcoholism is a complex disease believed to develop as the result of a combination of environmental influences and multiple underlying genes that may predispose some people toward the disorder. It is highly likely that an increased vulnerability to alcoholism results not only from DNA changes in the protein-coding regions of those genes, but also from more subtle variations in noncoding regions that affect when, where, and in what amount, the protein encoded by the gene is produced. The identification of underlying genetic differences could also provide valuable insight into the progression of alcohol tolerance, dependence, and toxic effects on the brain and could facilitate development of clinical treatments for alcoholism.

To comprehensively search for DNA changes that could contribute to alcohol-related disorders, sequences that represent gene-coding regions as well as those that influence the regulation and expression of genes need to be investigated. One hallmark of genomic analysis is its emphasis on techniques that identify and analyze large numbers of genes, rather than a few, at one time. One of these techniques is the use of high-density “gene chips” for gene expression studies. Similar to computer chips, gene chips are covered with minute amounts of DNA derived from different genes. They allow investigators to simultaneously compare the expression levels of thousands of genes between different individuals or under different environmental conditions.

Gene chips can be obtained through several commercial suppliers, as well as through the many academic gene chip facilities that have arisen to permit easy and inexpensive access to standard and custom sets of genes. Broadly speaking, there are three types of chip technologies (see table 1 for a list of related Web sites):

- *Affymetrix GeneChips*, available in a number of prefabricated varieties, use short, synthetic pieces of specific DNA sequences that are generated directly on the chips. They are used to evaluate mRNA expression in several different organisms, including humans, mice, and rats. Researchers can also order custom chips designed to examine specific genes of interest.
- *LifeArray^TM^ chips*, designed by IncyteGenomics, contain cDNA sequences corresponding to different human, mouse, or rat genes that are placed on small, specially coated glass slides.
- *“Exon” and “tiling” arrays*, generated by Rosetta Inpharmatics, employ a unique exon-specific approach that enables investigators to detect gene expression differences, including splicing variants, under a variety of different tissue or cell conditions at, potentially, the level of the entire genome (see Shoemaker et al. 2001).

As mentioned previously, all of these chips allow geneticists to investigate regulatory differences in the expression of thousands of genes at a time. Alcohol researchers are beginning to apply these methods in the study of both human alcoholism and alcohol-related phenotypes in mouse models.

## Applications to Alcohol Research

Several groups of alcohol researchers have begun exploring these state-of-the-art technologies to look for variations in gene expression related to the development of alcoholism or alcohol-related behaviors. Xu and colleagues (2001) used gene chips containing mouse DNA to identify 41 genes whose expression differed in the brains of 2 mouse strains called Inbred Short-Sleep (ISS) and Inbred Long-Sleep (ILS) mice, which have been genetically selected for differences in initial sensitivity to alcohol (McClearn and Kakihana 1981). Daniels and Buck (2002) have found evidence for differential regulation of gene expression during alcohol withdrawal in the hippocampal brain region of two mouse strains called C57BL/6J and DBA/2J. In this study, the expression of more than 100 genes, most of which fell into 6 major functional categories, differed substantially between the 2 strains.

These examples illustrate the importance of research involving animal models of alcohol-related phenotypes. Using animal models, researchers can carefully control and examine different conditions under which gene expression may vary, such as the extent of previous alcohol exposure and the developmental stage of the animals. Use of animal models also allows investigators to focus on specific brain regions, which may help identify the specific genes, proteins, and molecular mechanisms that underlie alcohol’s actions in the brain. Similar analyses can also be performed in cultured cells rather than in intact animals or tissues (Thibault et al. 2000).

Analysis of gene regulation in humans rather than in animal models is more challenging for numerous reasons. People who abuse alcohol vary substantially more in their genetic makeup than do inbred mouse strains. Furthermore, one cannot conduct the same experiments on humans as on animals simply because genetic material from the brains of humans is available only after their natural death, whereas animal samples can be obtained at various developmental stages. When differences in gene expression between alcohol-abusing and non-alcohol-abusing people are measured after death, however, it is difficult to determine whether they result from underlying genetic differences between the two groups or from the alcohol abuse. Despite their limitations, postmortem analyses may yield important results. For example, a recent study of gene expression in postmortem brain samples from alcoholics and matched control subjects revealed significant differences in the expression of certain genes that may be related to morphological differences in the brains of alcoholics and nonalcoholics, such as shrinkage of white matter (Lewohl et al. 2001).

The recent delineation of substantially complete sequences of both the human and mouse genomes has provided an important stimulus to genetic research in general as well as alcohol research in particular. Until now, the discovery of important disease-related gene sequence differences required that DNA sequencing be carried out by the investigating laboratory to find the relevant sequence change. The availability of extensive human and mouse genome sequence databases, however, may eliminate this requirement so that many such discoveries can now be made by computer, or “in silico.” For example, in the alcohol field, many mouse QTLs associated with certain alcohol effects were identified using the C57BL/6J and DBA/2J mice. Now, however, the draft genome sequences for these two strains are available, making it possible for investigators to select potential QTLs from the genome sequence databases of the two strains and, through computer analyses, search directly for any sequence differences. Although the genome sequence databases for these two strains still contain gaps, they cover a substantial part of the genome, allowing researchers to rapidly compare many QTL genes and identify differences. This approach has wide potential applicability and, in principle, could be used for QTL analysis in all mouse strains for which relatively complete genome sequences are available (Marshall et al. 2002).

## Conclusion

Ultimately, the identification of the genetic components and mechanisms that underlie the effects of alcohol on the brain must involve a convergence of efforts from investigators representing many fields, including behavior genetics, neurobiology, and molecular biology. Particularly because of the extensive new genomics resources that have been generated over the past decade, alcohol researchers now have unprecedented knowledge about the human and mouse genomes. The mining of such genome-based information offers the promise of uncovering many of the key genes and pathways that underlie complex genetic diseases, including alcoholism and alcohol abuse.

## Figures and Tables

**Table 1:** Resources for Genomic Analyses and Their Web Sites

**Public Precomputed Resources**	
UCSC Golden Path	http://genome.ucsc.edu/
NCBI Map Viewer	http://www.ncbi.nlm.nih.gov/
Ensembl	http://www.ensembl.org/
**Web-Based Annotation Systems**	
NIX	http://www.hgmp.mrc.ac.uk/Registered/Webapp/nix
RUMMAGE	http://gen100.imb-jena.de/rummage/index.html
GESTALT	http://bioinformatics.weizmann.ac.il/GESTALT
**Private Annotation Systems**	
Celera Genomics	http://www.celera.com
DoubleTwist™	http://www.doubletwist.com
**Gene Chip Providers**	
Affymetrix	http://www.affymetrix.com
LifeArray™ chips*	http://we.home.agilent.com
Rosetta Inpharmatics	http:www.rii.com

* Originally produced by IncyteGenomics, now available through Agilent.

**Table 2:** Genome Analysis Programs for Identifying Characteristic Features of Unknown DNA Sequences

Program Name	Type of Annotation	Program Location	Program Information
**Homology Searches**			
Repeats	Repeat Finder	Local	NCBI Repeats Database (http://repeatmasker.genome.washington.edu/cgi-bin/RM2_req.pl)
DbEST	Homology Search against EST database	Remote (Network-Client)	NCBI EST Database using Blastc13 (http://www.ncbi.nlm.nih.gov/BLAST)
Spliced ESTs	—	Local	Groups ESTs originated from a single clone
GenPept	Homology Search against NCBI protein database	Remote (Network-Client)	NCBI Protein Database using Blastc13 (http://www.ncbi.nlm.nih.gov/BLAST)
**Exon Prediction**			
Xpound	Gene Finder (rule-based)	Local	Software for exon trapping based on maximum likelihood methods (Kamb et al. 1995)
MZEF	Gene Finder (rule-based)	Local	Predicts putative internal protein coding exons in genomic DNA sequences, starts with potential exon and calculates posterior exon probability (Zhang 1997, 1998)
GrailEXP	Gene Finder (neural network)	Web Server	Incorporates EST homology searches and biological rules to model more complicated gene structures (http://compbio.ornl.gov/grailexp) (Xu et al. 1997)
GeneID	Gene Finder (neural network)	Local	Uses hierarchical structure to first identify splice sites, start and stop codons, then build exons, followed by scoring of exons and assembling gene structure (Parra et at. 2000)
Eukaryotic GeneMark.hmm	Gene Finder [Hidden Markov Model (HMM)]	Web Server	Relies upon an Inhomogeneous Markov Model approach combined with training data sets to predict genes (http://www.genemark.biology.gatech.edu/GeneMark/hum.cgi)
GENSCAN	Gene Finder (HMM)	Local	Uses general probabilistic model of gene structure assembling knowledge of basic transcriptional, translational, and splicing signals to predict exons (Burge and Karlin 1997)
Fgenesh	Gene Finder (HMM)	Local	Combines pattern recognition features with similarity searches of predicted exons against known protein databases (Solovyev and Salamov 1997)
**Other Analyses**			
ORF Finder	Open Reading Frame Finder	Web Server	http://www.ncbi.nih.gov/gorf/gorf.html
NNPP	Promoter Prediction	Local	Neural Network Promoter Prediction (NNPP) uses time-delay to predict promoters
Promoter-Inspector	Promoter Prediction	Web Server	Accurately predicts 43 percent of true promoters (http://genomatix.gsf.de/cgi-bin/promoterinspector/promoterinspector.pl) (Scherf et al. 2000)
GrailEXP CpG islands	CpG Island Finder	Web Server	Identifies regions containing short (200–2000bp) segments with a characteristic DNA composition (i.e., a GC content greater than 50 percent) that are commonly located near the starting regions of genes (http://compbio.ornl.gov/grailexp) (Xu et al. 1997)

NOTE: The results of these analyses can be entered into the Genotator program for a comprehensive analysis of unknown DNA sequences.

## Footnotes

1 An mRNA is a molecule generated in the nucleus, where the DNA is located, and serves as a template for the production of the corresponding gene product (i.e., protein), which occurs outside the nucleus.

2 These approaches are called neural network methods, rule-based systems, and Hidden Markov Models (HMMs).

3 EST stands for “expressed sequence tag,” which is a partial DNA sequence segment derived from randomly selected cDNAs.
